# Two-dimensional polymeric [Hg_4_(μ_2_-I)_6_I_2_(μ_2_-C_4_S_6_)]_*n*_

**DOI:** 10.1107/S1600536811006556

**Published:** 2011-02-26

**Authors:** Aurélien Hameau, Fabrice Guyon, Michael Knorr, Victoria P. Colquhoun, Carsten Strohmann

**Affiliations:** a Institut UTINAM UMR CNRS 6213, Université de Franche-Comté, 16 Route de Gray, La Bouloie, 25030 Besançon, France; b Anorganische Chemie, Technische Universität Dortmund, Otto-Hahn-Strasse 6, 44227 Dortmund, Germany

## Abstract

The title compound, poly[(μ_2_-2*H*,5*H*-1,3-dithiolo[4,5-*d*][1,3]di­thiole-2,5-dithione)hexa-μ_2_-iodido-diiodidotetra­mercury(II)], [Hg_4_I_8_(C_4_S_6_)]_*n*_, represents the first example of a coordination polymer assembled by the α,α-C_4_S_6_ dithione ligand. The Hg^II^ ions are four-coordinated in a distorted tetra­hedral geometry, the coordination demand being satisfied either by four bridging iodide ligands or by three iodide ligands (one terminal and two bridging) and a thio­carbonyl S atom. Due to the bridging nature of the dithione ligand, the coordination polymer has a two-dimensional structure, built up of undulated layers parallel to (001). There is an inversion center at the mid-point of the central C=C double bond.

## Related literature

For the synthesis and structure of the α,α-C_4_S_6_ ligand, see: Krug *et al.* (1977 ▶); Beck *et al.* (2006 ▶). For related studies on polymeric binary carbon sulfides, see: Galloway *et al.* (1994 ▶). For the synthesis and structures of coordination polymers with sulfur-rich ligands, see: Peindy *et al.* (2005 ▶); Hameau *et al.* (2006 ▶); Ndiaye *et al.* (2007 ▶); Guyon *et al.* (2008 ▶).
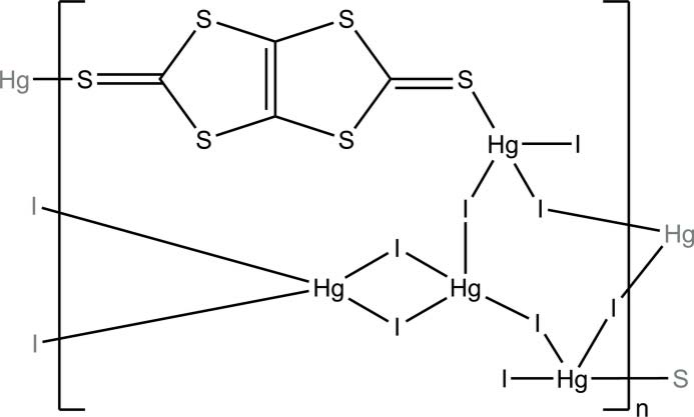


## Experimental

### 

#### Crystal data

[Hg_4_I_8_(C_4_S_6_)]
*M* *_r_* = 1028.98
Monoclinic, 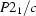

*a* = 8.5502 (6) Å
*b* = 11.2156 (8) Å
*c* = 13.4634 (9) Å
β = 91.343 (1)°
*V* = 1290.73 (16) Å^3^
*Z* = 4
Mo *K*α radiation
μ = 33.76 mm^−1^
*T* = 173 K
0.30 × 0.10 × 0.10 mm

#### Data collection

Bruker APEX CCD diffractometer
Absorption correction: multi-scan (*SADABS*; Bruker, 1999 ▶) *T*_min_ = 0.035, *T*_max_ = 0.133
24415 measured reflections
2543 independent reflections
2337 reflections with *I* > 2σ(*I*)
*R*_int_ = 0.086

#### Refinement

*R*[*F* ^2^ > 2σ(*F* ^2^)] = 0.039
*wR*(*F* ^2^) = 0.114
*S* = 1.03
2543 reflections
100 parameters
Δρ_max_ = 3.56 e Å^−3^
Δρ_min_ = −3.29 e Å^−3^

### 

Data collection: *SMART* (Bruker, 2001 ▶); cell refinement: *SAINT-Plus* (Bruker, 1999 ▶); data reduction: *SAINT-Plus*; program(s) used to solve structure: *SHELXS97* (Sheldrick, 2008 ▶); program(s) used to refine structure: *SHELXL97* (Sheldrick, 2008 ▶); molecular graphics: *ORTEP-3* (Farrugia, 1997 ▶); software used to prepare material for publication: *SHELXL97*.

## Supplementary Material

Crystal structure: contains datablocks I, global. DOI: 10.1107/S1600536811006556/fi2103sup1.cif

Structure factors: contains datablocks I. DOI: 10.1107/S1600536811006556/fi2103Isup2.hkl

Additional supplementary materials:  crystallographic information; 3D view; checkCIF report

## Figures and Tables

**Fig. 1.**
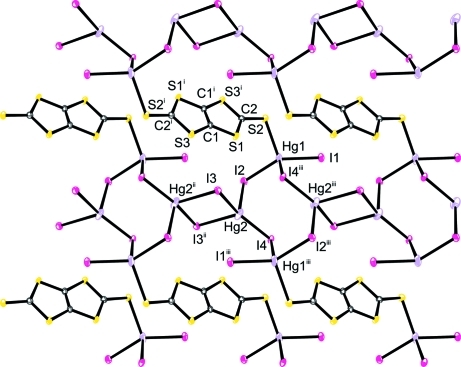
A view of the title compound along (001). Displacement ellipsoids are drawn at the 50% probability level. Symmetry operations: (i) -x, -y+2, -z+2; (ii) -x, -y+1, -z+2; (iii) -x+1, -y+1, -z+2.

## supplementary crystallographic information

### Comment

Molecular and polymeric binary carbon sulfides have been the subject of numerous studies (see for example Galloway *et al.*, 1994). In the context of our interest in using sulfur-rich ligands to synthesize coordination polymers (Peindy *et al.*, 2005; Hameau *et al.*, 2006; Ndiaye *et al.* 2007; Guyon *et al.* 2008), carbon sulfides and especially 1,3-dithiol*o*-(4,5-d)-1,3-dithiol-2,5-dithione (α,α-C_4_S_6_) appears attractive due to the presence of two potentially coordinating thiocarbonyl sulfur atoms. The α,α-C_4_S_6_ carbon sulfide compound, first prepared in 1977 (Krug *et al.*, 1977), reacts with HgI_2_ to afford the coordination polymer [Hg_4_I_8_(C_4_S_6_)]_n_ (1). As shown in Fig.1, the monomeric unit has a centrosymmetrical tetranuclear structure which is formed by one α,α-C_4_S_6_ ligand linking two Hg_2_I_4_ fragments with an inversion centre located at the mid-point of the central C═C bond. Each mercury(II) centre is arranged in a distorted tetrahedral manner. The Hg1 atom is coordinated by one terminal iodine atom (I1), two bridging iodine atoms (I2 and I4^iii^) and the sulfur of the thiocarbonyl function S2 whereas the coordination sphere of Hg2 involves only bridging iodo ligands (I2, I3, I3^ii^ and I4). Note however that the bridging contribution of I4 is weak since the Hg1^iii^-I4 distance (3.423 (1) Å) is quite long compared to that of Hg2—I4 (2.6497 (8) Å). The C═S bond of α,α-C_4_S_6_ is weakly affected by coordination of the sulfur atom on Hg1 (1.671 (10) Å *versus* 1.645 (2) in the free ligand, Beck *et al.*, 2006). The Hg1—S2 distance of 2.697 (3) Å is somewhat longer than that reported for 4,5-bis(methylthio)-1,3-dithiole-2-thione on HgI_2_ (Hameau *et al.*, 2006). The α,α-C_4_S_6_ ligands connect the inorganic chains built upon the alternance of 8-membered Hg_4_I_4_ and 4-membered Hg_2_I_2_ cycles to form a two-dimensional framework. Note that there are no S—S interactions inferior to the sum of the van der Waals radii of two S atoms in the solid state.

### Experimental

The α,α-C_4_S_6_ ligand was prepared as described previously (Beck *et al.*, 2006). To the α,α-C_4_S_6_ dithione (14 mg, 58 µmol) dissolved in 13.5 ml of a solvent mixture (toluene/acetonitrile/chlorobenzene in 2/1/1 ratio) was added upon stirring a solution of HgI_2_ (53 mg, 116 µmol) in toluene (10 ml). The resulting solution was refluxed for 0.2 h., then allowed to reach room temperature and filtered. Dark red crystals suitable for X-ray analysis were obtained by slow evaporation of the solution (yield 85%). IR (KBr): ν_C__═__S_ = 1036 cm^-1^.

### Refinement

The largest Fourier peak/hole (3.56 and –3.29 e/Å^3^, respectively) are found 0.95 and 0.68Å from Hg1 (see even extra table).

### Crystal data

**Table:**

[Hg_4_I_8_(C_4_S_6_)]	*F*(000) = 1728
*M**_r_* = 1028.98	*D*_x_ = 5.295 Mg m^−^^3^
Monoclinic, *P*2_1_/*c*	Mo *K*α radiation, λ = 0.71073 Å
Hall symbol: -P 2ybc	Cell parameters from 8987 reflections
*a* = 8.5502 (6) Å	θ = 2.4–26°
*b* = 11.2156 (8) Å	µ = 33.76 mm^−^^1^
*c* = 13.4634 (9) Å	*T* = 173 K
β = 91.343 (1)°	Needle, dark red
*V* = 1290.73 (16) Å^3^	0.30 × 0.10 × 0.10 mm
*Z* = 4	

### Data collection

**Table:**

Bruker APEX CCD diffractometer	2543 independent reflections
Radiation source: fine-focus sealed tube	2337 reflections with *I* > 2σ(*I*)
graphite	*R*_int_ = 0.086
ω scans	θ_max_ = 26.0°, θ_min_ = 2.4°
Absorption correction: multi-scan (*SADABS*; Bruker, 1999)	*h* = −10→10
*T*_min_ = 0.035, *T*_max_ = 0.133	*k* = −13→13
24415 measured reflections	*l* = −16→16

### Refinement

**Table:**

Refinement on *F*^2^	0 restraints
Least-squares matrix: full	Primary atom site location: structure-invariant direct methods
*R*[*F*^2^ > 2σ(*F*^2^)] = 0.039	Secondary atom site location: difference Fourier map
*wR*(*F*^2^) = 0.114	*w* = 1/[σ^2^(*F*_o_^2^) + (0.077*P*)^2^ + 7.1937*P*] where *P* = (*F*_o_^2^ + 2*F*_c_^2^)/3
*S* = 1.03	(Δ/σ)_max_ = 0.001
2543 reflections	Δρ_max_ = 3.56 e Å^−^^3^
100 parameters	Δρ_min_ = −3.29 e Å^−^^3^

### Special details

**Table:**

Geometry. All e.s.d.'s (except the e.s.d. in the dihedral angle between two l.s. planes) are estimated using the full covariance matrix. The cell e.s.d.'s are taken into account individually in the estimation of e.s.d.'s in distances, angles and torsion angles; correlations between e.s.d.'s in cell parameters are only used when they are defined by crystal symmetry. An approximate (isotropic) treatment of cell e.s.d.'s is used for estimating e.s.d.'s involving l.s. planes.
Refinement. Refinement of *F*^2^ against ALL reflections. The weighted *R*-factor *wR* and goodness of fit *S* are based on *F*^2^, conventional *R*-factors *R* are based on *F*, with *F* set to zero for negative *F*^2^. The threshold expression of *F*^2^ > σ(*F*^2^) is used only for calculating *R*-factors(gt) *etc*. and is not relevant to the choice of reflections for refinement. *R*-factors based on *F*^2^ are statistically about twice as large as those based on *F*, and *R*- factors based on ALL data will be even larger.

### Fractional atomic coordinates and isotropic or equivalent isotropic displacement parameters (Å^2^)

**Table:**

	*x*	*y*	*z*	*U*_iso_*/*U*_eq_	
C1	0.0263 (12)	0.9542 (9)	1.0288 (7)	0.029 (2)	
C2	0.2439 (12)	0.9942 (9)	0.9076 (8)	0.033 (2)	
Hg1	0.51629 (6)	0.77041 (5)	0.83365 (5)	0.05585 (19)	
Hg2	0.22624 (7)	0.46970 (6)	0.97826 (5)	0.0672 (2)	
I1	0.82359 (9)	0.77867 (6)	0.83985 (5)	0.0374 (2)	
I2	0.25603 (8)	0.64475 (6)	0.79810 (5)	0.0372 (2)	
I3	0.08558 (9)	0.60123 (6)	1.11662 (5)	0.03616 (19)	
I4	0.45656 (8)	0.32667 (6)	0.92556 (5)	0.03444 (19)	
S1	0.2076 (3)	0.8973 (2)	1.0033 (2)	0.0374 (6)	
S2	0.4123 (3)	0.9968 (3)	0.8465 (2)	0.0427 (6)	
S3	−0.0987 (3)	0.9043 (2)	1.1185 (2)	0.0363 (6)	

### Atomic displacement parameters (Å^2^)

**Table:**

	*U*^11^	*U*^22^	*U*^33^	*U*^12^	*U*^13^	*U*^23^
C1	0.025 (5)	0.035 (5)	0.028 (5)	−0.001 (4)	0.003 (4)	0.002 (4)
C2	0.031 (5)	0.029 (5)	0.039 (5)	−0.002 (4)	0.003 (4)	−0.001 (4)
Hg1	0.0325 (3)	0.0672 (4)	0.0682 (4)	−0.0029 (2)	0.0078 (2)	0.0044 (3)
Hg2	0.0477 (4)	0.0668 (4)	0.0881 (5)	0.0157 (3)	0.0243 (3)	−0.0110 (3)
I1	0.0303 (4)	0.0452 (4)	0.0369 (4)	−0.0012 (3)	0.0048 (3)	−0.0032 (3)
I2	0.0332 (4)	0.0424 (4)	0.0359 (4)	−0.0019 (3)	0.0013 (3)	0.0038 (3)
I3	0.0351 (4)	0.0365 (4)	0.0369 (4)	−0.0014 (3)	0.0029 (3)	−0.0047 (3)
I4	0.0305 (4)	0.0404 (4)	0.0326 (4)	0.0048 (3)	0.0041 (3)	−0.0024 (3)
S1	0.0310 (14)	0.0406 (14)	0.0410 (14)	0.0074 (11)	0.0101 (11)	0.0073 (11)
S2	0.0350 (15)	0.0365 (13)	0.0576 (17)	0.0020 (11)	0.0205 (13)	0.0038 (12)
S3	0.0339 (14)	0.0372 (13)	0.0383 (13)	0.0051 (10)	0.0128 (11)	0.0067 (11)

### Geometric parameters (Å, °)

**Table:**

C1—C1^i^	1.36 (2)	Hg1—S2	2.697 (3)
C1—S1	1.718 (11)	Hg2—I4	2.6496 (9)
C1—S3	1.724 (11)	Hg2—I3	2.6828 (9)
C2—S2	1.675 (11)	Hg2—I3^ii^	3.0353 (10)
C2—S3^i^	1.715 (11)	Hg2—I2	3.1357 (10)
C2—S1	1.720 (11)	I3—Hg2^ii^	3.0353 (10)
Hg1—I1	2.6285 (9)	S3—C2^i^	1.715 (11)
Hg1—I2	2.6678 (9)		
			
C1^i^—C1—S1	117.1 (11)	I4—Hg2—I3^ii^	112.31 (3)
C1^i^—C1—S3	116.3 (11)	I3—Hg2—I3^ii^	91.87 (3)
S1—C1—S3	126.6 (6)	I4—Hg2—I2	95.60 (3)
S2—C2—S3^i^	121.0 (6)	I3—Hg2—I2	103.81 (3)
S2—C2—S1	123.4 (6)	I3^ii^—Hg2—I2	85.70 (3)
S3^i^—C2—S1	115.5 (6)	Hg1—I2—Hg2	105.93 (3)
I1—Hg1—I2	148.57 (3)	Hg2—I3—Hg2^ii^	88.13 (3)
I1—Hg1—S2	107.19 (7)	C1—S1—C2	95.4 (5)
I2—Hg1—S2	103.53 (7)	C2—S2—Hg1	107.7 (4)
I4—Hg2—I3	150.11 (4)	C2^i^—S3—C1	95.7 (5)

Symmetry codes: (i) −*x*, −*y*+2, −*z*+2; (ii) −*x*, −*y*+1, −*z*+2.

### Table 1 Final difference electron densities

**Table:**

Qx	nearest atom	distance	value
-Q1	Hg1	0.68	-3.29
Q1	Hg1	0.951	3.56
Q2	Hg2	0.994	1.67
Q3	Hg2	0.770	1.49
Q4	I3	0.797	1.07
Q5	Hg1	1.256	0.92
Q6	I3	0.688	0.92
